# Evaluation of Outpatient Access to the Greek Health System Considering the Constraints of the COVID-19 Pandemic

**DOI:** 10.7759/cureus.36704

**Published:** 2023-03-26

**Authors:** Georgios F Fragkiadakis, Anastasia Tsatinian

**Affiliations:** 1 Social Sciences, Hellenic Open University, Chania, GRC; 2 Social Sciences, Hellenic Open University, Xanthi, GRC

**Keywords:** covid-19, health policy, outpatient, public health, patient satisfaction

## Abstract

Background

The COVID-19 pandemic fundamentally changed the way healthcare is delivered in most healthcare systems around the world. It is now known that, in addition to the medical and economic impact of the pandemic on societies, there is another unmet medical need due to the difficulties and barriers that have existed and may still exist in the provision of health services at the primary care level in public hospitals. In Greece, there appears to have been a similar problem in citizens' access to health care in the country's public hospitals, which negatively affected the satisfaction of outpatients and significantly prevented them from receiving the medical care they needed.

Methods

This study relied on two international questionnaires; the Visit Specific Satisfaction (VSQ-9), an instrument measuring patients' satisfaction with their visit to the physician, and the Patient Satisfaction Questionnaire Short-Form (PSQ-18), consisting of 18 questions measuring both satisfaction and dissatisfaction. The questionnaires were collected electronically between 01.03.22 and 20.03.22 from two hundred and three outpatient residents of the region of Eastern Macedonia and Thrace in Greece.

Results

The results of the study show that satisfaction of hospital outpatient department users is positively influenced by both access to medical care after the last visit (p=0.008 < 0.05) and frequency of visits (Pearson correlation coefficient=0.178, p=0.012). In addition, lower satisfaction with access to care was found among participants with the lowest income (p=0.010) and those with a chronic illness (p=0.002), which was attributed to pandemic limitations in access to health care services in public hospital outpatient departments. Regarding the overall satisfaction of participants, 40.9% were dissatisfied, and 32.5% were dissatisfied with specific hospital services.

Conclusions

It was found that patients were hindered from accessing medical care in the hospital due to the restrictions caused by the pandemic. This seemed to cause problems both in accessing a specialist and in making appointments. Half of the outpatients in the sample reported having difficulty communicating with the hospital to make an appointment or to access medical services in general. A relationship was also found between patient satisfaction and the quality of services provided, in terms of medical services offered, such as their availability, and patient satisfaction with the appropriate information they receive from physicians during the pandemic period. The study also found that long-term care hospitals need to improve patient satisfaction with existing medical services.

## Introduction

Quality of healthcare is one of the most frequently cited principles of health policy and is currently high on the agenda of policymakers at national, European, and international levels. In the past, the process of quality assessment was conducted without considering the patient' perspective. Today, more emphasis is placed on patient feedback, which is important for several reasons. First, satisfactory quality of healthcare services is related to issues such as adherence to medical guidelines or reuse of services. The Institute of Medicine (IOM), for example, defines quality of care as "doing the right thing, at the right time, in the right way, for the right person, and with the best possible outcomes". In addition, public and private initiatives to develop healthcare measures have been guided by several important quality assessment factors. One of the most influential concepts is the framework established by the IOM, which includes six goals for the healthcare system. These concepts - safety, effectiveness, patient-centeredness, timeliness, efficiency, and equity - are considered essential to quality [1].

Patient satisfaction in health care affects all healthcare systems in the world, even if it is classified in a spectrum of ambiguity and subjectivity. Nevertheless, the influence of patient feedback in relation to the management of healthcare services is an important factor in identifying problems and difficulties encountered in healthcare facilities, as well as quality gaps in service delivery [2,3]. The integration of quality concepts in the management of healthcare systems is increasingly classified as a key tool for their smooth functioning [4,5].

At the national level, the quality of health care can be improved through continuous monitoring of health services and patient satisfaction in order to take appropriate action. From a policy perspective, soliciting patient opinions is a democratic necessity that is a central feature of the harmonious functioning of modern societies. Moreover, patient participation restores trust in health services, ultimately improving their performance and contributing to social cohesion [6, 7]. From a sociological point of view, patient participation in the therapeutic strategy automatically strengthens the cooperative dimension of care by weakening the antiquated paternalistic approach that used to be a main feature of the therapeutic relationship. From a legal point of view, patient satisfaction has become a legal obligation in many countries in the last decades. In France, for example, it has been enshrined in the legal code since the reform of the health care system in 1966 [8].

Service quality assessment can help managers of healthcare facilities identify patient needs and service delivery problems to develop solution programs that improve both financial performance and patient satisfaction [9, 10]. According to O'Connor et al., there are two types of patient expectations: the potential medical services they will receive and those they desire [11]. Managers of healthcare facilities need to know these because unmet expectations lead to low satisfaction rates [12]. In addition to the previous findings, there are still important parameters that should be taken into account and that are examined in the present study as well as in the previous studies. The correlation test between service quality and patient satisfaction showed a strong and significant correlation between service quality and overall satisfaction (p<0.001, r=0.73). Among the dimensions of service quality, physician consultation was strongly associated with overall satisfaction, followed by perceived service costs and patient information [13].

To measure patients' satisfaction with their hospital care, several instruments examine their experiences in this regard. Some of them are the Visit Specific Satisfaction Questionnaire (VSQ-9), developed by the American Medical Group Association; the Patient Satisfaction Questionnaire Short-Form (PSQ-18); and the Client Satisfaction Questionnaire (CSQ-18/CSQ-8), which measures patients' satisfaction with medical care [14]. In addition, the Patient Experience Questionnaire aims to assess the relationship between patient experience, aspects of health care, and patient characteristics [15]. Finally, the SERVQUAL provides the opportunity to measure services in five dimensions. However, it has been found to have several methodological shortcomings, with the most serious problems being found in the assessments [16].

Impact of COVID-19 on healthcare in Greece

In Greece, the COVID-19 pandemic brought significant changes to the healthcare system, with 13 hospitals declared major referral hospitals for COVID-19 cases. Clinics were closed and wards evacuated to establish negative pressure units and intensive care units for COVID-19 cases, while scheduled outpatient appointments were canceled. Scheduled surgeries were canceled and only emergency surgeries were performed while all attention was focused on managing the pandemic without considering the health needs of the population. The pandemic highlighted the limited resources and gaps in the NHS [17].

The increased need for medical care for COVID-19 cases during the pandemic also led to a large demand for healthcare workers. Greece strengthened its healthcare system with more than 6,800 new hires, including medical, nursing, paramedical, and other personnel. Staff shortages continued in public hospitals, and private physicians were allowed to work in public hospitals in April 2020. Redistribution of health care to fill beds with COVID-19 patients, staff shifts from primary care to hospitals, and additional overtime by healthcare workers were just some of the additional demands the pandemic placed on the healthcare system [18].

The COVID-19 pandemic also radically changed the way cases were treated in the hospital outpatient departments of the Greek health system. Many of the regular outpatient clinics discontinued or restricted their outpatient appointments, depending on the medical specialty. The role of the outpatient clinics was mainly taken over by the emergency department, which, by its nature and the objectives of the hospitals, is mainly and in some cases exclusively responsible for medical emergencies. This phenomenon prevailed in most hospitals in the country due to the decisions of the central political leadership to limit visits to Greek public hospitals because of the high caseload. The difficulty for outpatients to access medical services during the pandemic period has also been described in other countries [19, 20]. In addition, limited access to routine outpatient care during the lockdown has highlighted the risk of underestimating the consequences of chronic diseases if adequate follow-up is not provided [21].

Another study found that inpatient visits increased because of the need for medical follow-up after hospitalization or surgery, which somewhat offset the decrease in regular outpatient hospital visits. An important factor in the variability of visits was the use of telemedicine care or remote diagnosis in some specific cases. The results of this particular survey showed a transition from in-person visits before the pandemic to predominantly virtual care during the pandemic [22]. However, the extent to which in-person visits decreased and were replaced by virtual health visits remains unclear [23].

The aim of this study was to determine the level of satisfaction of outpatients of a public hospital in the province of the Greek Health System in relation to the availability of medical services during the period COVID-19. Subsequently, other factors related to health service users were investigated, such as waiting times, correct medical information, and adequate examination times.

## Materials and methods

Data collection instruments and population

The collection of the questionnaires was carried out entirely electronically to the citizens of the region of Eastern Macedonia and Thrace. The completion of the questionnaires lasted from 01/3/2022 to 20/3/2022. It was indicated that participation was voluntary and that the anonymity of the personal data of the sample was guaranteed.

The survey was conducted with two international questionnaires prepared in Greek according to the guidelines of RAND Health Care [24,25]. One was the Visit Specific Satisfaction (VSQ-9), an instrument developed by the American Medical Group Association in 2000 to measure patient satisfaction with the physician visit, and the other was the Patient Satisfaction Questionnaire Short-Form (PSQ-18), which consists of 18 questions, some of which measure satisfaction and others dissatisfaction.

The VSQ-9 examines appointment scheduling and waiting time, accessibility, telephone communication, waiting for an examination, the amount of time the physician spends on the examination, the physician's explanations of the health condition and treatment, the physician's professional skills and demeanor, and finally the overall impression of the visit. The scale is five-point and ranges from one to five, with a score of one corresponding to the "poor" option and a score of five corresponding to the "excellent" option. The score is obtained when the values are converted to percentile values, with zero corresponding to the "poor" option and one corresponding to the "excellent" option. The responses to the nine questions should then be summed to obtain a VSQ-9 percentile score for each person [24].

The PSQ-18 also examines patient satisfaction and focuses mainly on seven dimensions: overall satisfaction, technical quality, physician behavior, physician communication, financial, physician time spent with patients, and access to care. The scale is five-point, ranging from one for "strongly agree" to five for "strongly disagree." The score is the sum of the individual scores for the questions that make up each dimension after reverse coding the responses where appropriate. The score reflects the patient's satisfaction with each dimension, with a high score reflecting a higher level of satisfaction [25].

Statistical analysis

Counts and percentages were used to describe categorical measures such as gender and employment status, whereas means and standard deviations were used for continuous measures such as questionnaire scores. Questionnaire reliability was assessed using Cronbach's α-index. Mann-Whitney (Kruskal-Wallis) tests were conducted for two independent samples to differentiate VSQ-9 questionnaire scores by characteristics or other parameters expressed in categories after Shapiro-Wilk normality tests were conducted. Accordingly, two independent-sample t-tests were conducted to differentiate PSQ-18 questionnaire scores by characteristics or other parameters expressed in two categories. At the same time, the influence of marital status and other categories on more than two options was evaluated using the analysis of variance criterion. Finally, to study the dependence of two quantitative variables, Pearson's correlation coefficient was used. The analysis was performed using Statistical Product and Service Solutions (SPSS) v23.0 software (IBM Inc., Armonk, New York), and the significance level was set at 0.05 in all cases.

## Results

Demographic data of the study population 

As shown in Table 1, 203 people participated in the survey, including 54 men (proportion 26.7%) and 148 women (proportion 73.3%). Their ages varied from 19 to 75 years, with an average of 35 years. In terms of education level, 85 people had a college/teaching degree (41.9%). We note that the sample is mainly composed of citizens who are insured and employed in a fund. In addition, a fairly high percentage of the sample has completed higher education. The reported income of the respondents is up to 20 thousand euros.

**Table 1:** Demographic data and individual characteristics of the participants

Variables		N	%
Gender	Male	54	26.7%
	Female	148	73.3%
Marital status	Single	99	48.8%
	Married	92	45.3%
	Divorced	10	4.9%
	Widowed	2	1.0%
Education level	Primary education	2	1.0%
	Secondary education	4	2.0%
	Higher education	72	35.5%
	University degree	85	41.9%
	Master’s degree/PhD	40	19.7%
Employment status	Employee	173	85.2%
	Unemployed	25	12.3%
	Pensioners	5	2.5%
Type of health insurance	National health insurance	155	76.4%
	Private insurance	11	5.4%
	Other public insurance	34	16.7%
	No insurance	3	1.5%
Annual net earnings	0-10.000	92	45.3%
	10.001-20.000	84	41.4%
	20.001-30.000	24	11.8%
	30.001-40.000	2	1.0%
	>40.001	1	0.5%

Distribution of the dimensions of the PSQ-18 questionnaire according to demographic characteristics

Regarding the correlation between the demographic characteristics and the level of satisfaction in the VSQ-9, no statistically significant relationship was found. However, in PSQ-18 (Table 2), the type of employment of the participants was found to influence the dimensions of medical behavior and financial perspective. Specifically, we see that in the dimension of medical behavior, the scores for retirees were higher than for the other participants (p=0.043), i.e., retirees reported being more satisfied with medical behavior. Also, in the dimension of financial aspects, the values for professionals predominated (p=0.029), i.e., professionals indicated to be more satisfied with financial aspects than other respondents. The net annual income of the participants also influenced the values in the dimension of access to medical care in the sense that lower values prevailed for participants with an income of 0-10,000 euros per year (p=0.010), i.e., participants in this income category reported being less satisfied with access to medical care than others.

**Table 2:** Distribution of dimensions of the PSQ-18 questionnaire in relation to the type of employment and annual net earnings of participants PSQ-18 - Patient Satisfaction Questionnaire Short-Form

Variables		N	Mean	Standard deviation	Standard error	95% CI		p-value
						Lower limit	Upper limit	
Medical behavior	Employed	173	3.1908	0.54562	0.04148	3.1089	3.2726	0.043
	Unemployed	25	3.12	0.6	0.12	2.8723	3.3677	
	Pensioners	5	3.8	0.67082	0.3	2.9671	4.6329	
	Total	203	3.197	0.5612	0.03939	3.1194	3.2747	
Financial aspects	Employed	173	3.2977	0.75836	0.05766	3.1839	3.4115	0.029
	Unemployed	25	2.86	0.75719	0.15144	2.5474	3.1726	
	Pensioners	5	3.1	1.29422	0.57879	1.493	4.707	
	Total	203	3.2389	0.78243	0.05492	3.1306	3.3472	
Access to healthcare	0-10.000	92	2.4783	0.70113	0.0731	2.3331	2.6235	0.01
	10.001-20.000	84	2.7341	0.724	0.07899	2.577	2.8912	
	20.001-30.000	24	2.8333	0.62167	0.1269	2.5708	3.0958	
	30.001-40.000	2	2.8333	1.17851	0.83333	-7.7552	13.4218	
	Total	203	2.6223	0.72189	0.05067	2.5224	2.7222	

Results related to participants' experiences with the hospital

It was found that participants who had or had not recently visited an outpatient clinic (p=0.008 < 0.05) and frequency of visits (Pearson=0.178, p=0.012) influenced questionnaire scores. When the surveyed sample was asked a clarifying question about reasons for waiting for a doctor's appointment, 54% of responses related to pandemic constraints. In terms of VSQ-9 satisfaction (Table 3) of those who visited outpatient clinics, it was also found that the largest percentage of patients were in the "moderate" or "good" range for almost all questions on the five-point scale, with the exception of the questions about wait times, where the highest percentages were concentrated on the fair and poor scales. When asked how long they waited for an appointment, 23.2% responded poorly, and 49.3% responded moderately. 

**Table 3:** Distribution of participant responses to the VSQ-9 patient satisfaction questionnaire VSQ-9 - Visit Specific Satisfaction Questionnaire

Variables	Poor		Fair		Good		Very Good		Excellent	
	N	%	N	%	N	%	N	%	N	%
How long you waited to get an appointment	47	23.2%	100	49.3%	40	19.7%	12	5.9%	4	2.0%
Convenience of the location of the office	5	2.5%	33	16.3%	109	53.7%	41	20.2%	15	7.4%
Getting through to the office by phone	32	15.8%	101	49.8%	50	24.6%	17	8.4%	3	1.5%
Length of time waiting at the office	59	29.1%	86	42.4%	46	22.7%	8	3.9%	4	2.0%
Time spent with the physician/health care professional you saw	8	3.9%	75	36.9%	89	43.8%	23	11.3%	8	3.9%
Explanation of what was done for you	16	7.9%	67	33.0%	77	37.9%	33	16.3%	10	4.9%
Technical skills of the physician/health care professional you saw	16	7.9%	56	27.6%	79	38.9%	34	16.7%	18	8.9%
The personal manner (courtesy, respect, sensitivity, friendliness) of the person you saw	18	8.9%	86	42.4%	69	34.0%	23	11.3%	7	3.4%
The visit overall	12	5.9%	66	32.5%	85	41.9%	29	14.3%	11	5.4%

In addition, the results of the PSQ-18 questionnaire (Table 4) show that those who have a chronic disease have lower scores on access to health care; that is, they are less satisfied with access to health care than those who do not have a chronic disease. Regarding satisfaction with the PSQ-18, the highest percentages for several questions were on the middle scale, and the next highest percentages were on the second and fourth of the five-point scale. More specifically, in the general satisfaction dimension, 40.9% of the participants agreed that they were satisfied with the medical care they received, while 32.5% indicated that they were dissatisfied with certain things about the medical care they received in the outpatient clinics.

**Table 4:** Distribution of the seven dimensions of the PSQ-18 questionnaire in relation to the question "Have you visited the hospital's outpatient clinics in the last year?" PSQ-18 - Patient Satisfaction Questionnaire Short-Form

Have you visited the hospital's outpatient clinics in the last year?		N	Mean	Standard deviation	Standard error of the mean	p-value
General satisfaction	No	89	2.5056	.72101	.07643	.014
	Yes	114	2.7851	.85178	.07978	
Technical quality	No	89	2.8202	.65060	.06896	.087
	Yes	114	2.9803	.66531	.06231	
Interpersonal manner	No	89	3.1854	.53503	.05671	.795
	Yes	114	3.2061	.58299	.05460	
Communication	No	89	3.2921	.63429	.06723	.696
	Yes	114	3.3289	.68753	.06439	
Financial aspects	No	89	3.0899	.77812	.08248	.016
	Yes	114	3.3553	.76922	.07204	
Time spent with doctor	No	89	2.6910	.65931	.06989	.849
	Yes	114	2.7105	.76971	.07209	
Accessibility and convenience	No	89	2.4831	.69087	.07323	.015
	Yes	114	2.7310	.72986	.06836	

In the dimension of the questionnaire that includes technical quality, 36.5% responded negatively to the question of whether outpatient clinics provide comprehensive medical care, while to the question of whether there is doubt about the accuracy of the medical diagnosis received from the medical staff, 28.6% of patients responded positively or affirmatively. In addition, 36% of survey participants reported difficulty in getting an appointment at the outpatient clinic in a timely manner, and 28.1% reported not receiving treatment when they needed it.

Outcomes related to participants' access to medical services based on COVID-19 limitations

**Figure 1:**
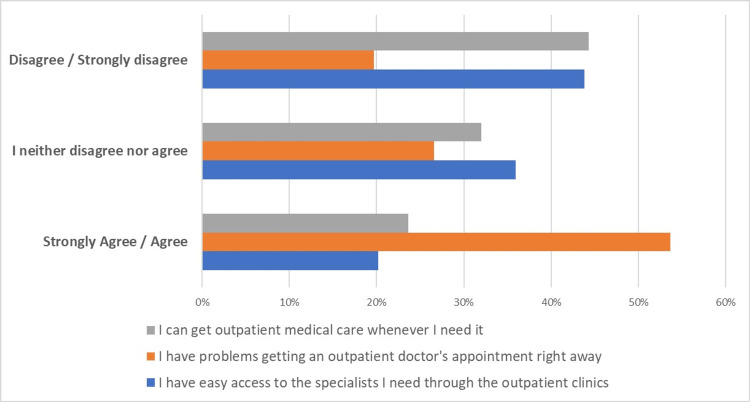
Distribution of participants' answers to the questions of the dimension "access to medical care"

In this section, the survey focused on the potential barriers patients face in accessing health care in hospital outpatient departments. The questions they were asked were adapted accordingly and included questions related to limitations in accessing hospital care due to the pandemic. The questions in Figure 1 were preceded by a statement by the researchers that they were to be answered based on their experience in the last year in which the COVID-19 restrictions were applied. Thus, patients answered the questions considering all the limitations related to easy access to physicians on-site (rather than by phone), difficulties in making appointments at outpatient clinics, and finally, whether they could receive medical services whenever they were needed. The results of the patients' responses show that about 43% of them had difficulties in seeing a specialist during the pandemic, while 53% of the respondents agreed that it was difficult to get an appointment with a doctor in the outpatient clinic. In addition, 32% of patients reported that they could not access medical services when they needed them for their health.

## Discussion

Benchmarking with corresponding surveys on the duration of COVID-19

The COVID-19 pandemic has revealed several problems in patient-physician interactions in various healthcare systems. Several countries have focused on limiting the problems in meeting the medical needs of non-ill patients by providing solutions mainly with remote patient care. Specifically, the Centre Hospitalier de l'Université de Montréal (CHUM), a university hospital in Montreal in Québec, in Canada, has developed a platform that includes two innovative technological applications that were developed before the pandemic for online consultations and remote patient monitoring, among other things. During the pandemic, these applications were adapted for remote monitoring of patients with COVID-19 [26-27]. In Greece, there is the possibility of developing similar platforms in public hospitals to care for patients remotely, provided the necessary human and financial resources are available. This should of course be preceded by decisions by the relevant bodies of the Ministry of Health to institutionalize this as a service provided by health facilities, and followed by regulatory decisions allowing the use of new technologies and permission for doctors to provide medical advice at a distance. Furthermore, one study found high satisfaction rates among patients and their caregivers with the use of teleconsultation services recently introduced in hospital outpatient departments during the COVID-19 pandemic. The services in question are something new for health systems to support patient demand for health services, especially for patients with long-term conditions [28].

Despite a dramatic decrease in physician visits before, during, and after the lockdown, most patients did not feel that access to pain physicians and medications was significantly affected. However, higher levels of anxiety and depression were noted, which appeared to be influenced by several factors related to quality of life and medications received, but also appeared to affect patients' overall satisfaction with the services they received. In addition, we can conclude that the effects of the lockdown affected patients more than the pandemic itself. Most patients' perception of pain was not affected by the pandemic and was only slightly affected by the lockdown. One study highlighted both the need for and the complications of implementing telemedicine for Greek patients with chronic pain [29]. Another study concluded that health services need to be improved during this period COVID-19 so that we can contribute to better patient confidence, which has a positive impact on health outcomes [30]. 

Benchmarking with similar surveys to assess patient satisfaction

According to corresponding studies [31-34], problems with organization and bureaucracy seem to have a significant impact on the negative evaluation of the services offered. The study by Pierrakos et al [32] found that 40% of patients found it difficult to choose the day and time, and 45% that the time between when they wanted to book the appointment and the day of the visit was long. In addition, 45% of patients felt the waiting time in the waiting room was long enough and 86% were satisfied with the time it took the doctor to complete the exam, although 48% reported that the exam time was < 10 minutes. According to our research, problems with patient wait times and appointment length were also observed in several other hospitals in the country, as shown by other studies. On the other hand, a study by Baker et al [31] showed that patient satisfaction increases as long as there is a continuous communication relationship. In the present study, those who had visited outpatient clinics more frequently in the past year had higher scores, indicating greater satisfaction with the VSQ-9 questionnaire. It appears that the pandemic's restrictive policies on managing patient visits to hospital outpatient departments had a negative impact on patient satisfaction. In particular, patients gave poor or moderate ratings for services related to the process and waiting time for an appointment with a doctor.

In a survey conducted among regular outpatients of the General Hospital of Larissa in Greece, high satisfaction rates were found with various services provided by the hospital. Specifically, 79.5% were satisfied with medical care, 67% with administrative services, 75.9% with nursing services, and 59.1% of visitors were satisfied with facilities [34]. In contrast to the results of the present work, in the dimension of "technical quality", 36.5% of respondents were dissatisfied with the adequacy of hospitals to provide comprehensive medical care. High satisfaction rates were also found among medical staff, although in comparison, only 37.9% declared to be fully satisfied. In a survey conducted in four hospitals in Cyprus, quite high satisfaction rates were found for medical care, courtesy of staff, doctor's instructions, and duration of the examination, even if it was less than 10 minutes. The results showed no correlations in terms of gender, education level, and frequency of visits. Of the participants, 61.8% were satisfied with outpatient services, with satisfaction increasing with age and better health status. Nine out of 10 patients reported being satisfied with the physician's behavior, and four out of 10 patients stated that they were very dissatisfied with the waiting time from the first attempt to make an appointment to its completion. Finally, six out of 10 patients reported waiting more than a month for their examination, and 23.5% >3 months. Long wait times for appointments and waiting room examinations have been associated with a decrease in overall patient satisfaction, as longer wait times increase dissatisfaction [35]. Increasing dissatisfaction with long waiting times is one of the main problems faced by hospitals and contributes to low patient satisfaction, as shown by most studies [36-38]. Finally, the number of physicians and nurses in the health care system proves that there is a strong correlation between the level of patient satisfaction and the number of hospital beds, nurses, and physicians per 100,000 population, with the latter being the largest contributor [2]. 

Limitations

The survey was conducted by filling out the questionnaire electronically, which limited the participation of patients because of their low knowledge of using electronic media, especially in the older age groups. In addition, we believe that the electronic transmission influenced the age group of the sample because the elderly did not have access to electronic media or did not have the aforementioned knowledge that would have helped them to complete the electronic participation form. An important factor that was not taken into account and that we believe would explain some of the results of the study is that the participants were not asked to indicate the health problem they had or the medical specialty they needed to consult. Finally, we must point out that it would be useful to compare the results in corresponding health districts or hospitals in the same period to see if the results of our study are confirmed in other areas with public hospitals.

## Conclusions

The aim of the present study was to assess the satisfaction of visitors to a public hospital during the pandemic and to investigate the possible obstacles faced by patients in receiving adequate medical care in hospital outpatient departments. The ratings given by patients generally indicate the presence of barriers to accessing medical services, and it seems that their satisfaction is significantly related to the number of visits they were able to perceive in the past year. The results of this study also indicate that chronically ill patients experience difficulties in accessing care. Given the low patient satisfaction with waiting and examination times, it would also be useful to consider and implement measures to provide medical care without long waiting times by improving appointment management and medical examination times.

An important factor in improving patient satisfaction seems to be the possibility of providing medical services remotely, especially in times of a pandemic, especially in provincial hospitals, to reduce unmet medical needs due to limited access or availability of doctors. In summary, continuous assessment of facilities is considered essential for the continuous improvement of the quality of health services.
